# Optical and Photoconversion Properties of Ce^3+^-Doped (Ca,Y)_3_(Mg,Sc)_2_Si_3_O_12_ Films Grown via LPE Method onto YAG and YAG:Ce Substrates

**DOI:** 10.3390/ma18153590

**Published:** 2025-07-30

**Authors:** Anna Shakhno, Vitalii Gorbenko, Tetiana Zorenko, Aleksandr Fedorov, Yuriy Zorenko

**Affiliations:** 1 Department of Physics, Kazimierz Wielki University in Bydgoszcz, 85-090 Bydgoszcz, Poland; gorbenko@ukw.edu.pl (V.G.); tzorenko@ukw.edu.pl (T.Z.); 2 Mechantronic Department, Kazimierz Wielki University in Bydgoszcz, 85-074 Bydgoszcz, Poland; 3 Institute for Single Crystal, National Academy of Science of Ukraine, Nauky Ave., 60, 61000 Kharkiv, Ukraine

**Keywords:** Ca-Mg-Si-based garnets, Ce^3+^ dopant, liquid phase epitaxy, single crystalline films, crystal substrates, luminescence, phosphor converters

## Abstract

This work presents a comprehensive study of the structural, luminescent, and photoconversion properties of epitaxial composite phosphor converters based on single crystalline films of Ce^3+^-activated Ca_2−x_Y_1+x_Mg_1+x_Sc_1−x_Si_3_O_12_:Ce (x = 0–0.25) (CYMSSG:Ce) garnet, grown using the liquid phase epitaxy (LPE) method on single-crystal Y_3_Al_5_O_12_ (YAG) and YAG:Ce substrates. The main goal of this study is to elucidate the structure–composition–property relationships that influence the photoluminescence and photoconversion efficiency of these film–substrate composite converters, aiming to optimize their performance in high-power white light-emitting diode (WLED) applications. Systematic variation in the Y^3+^/Sc^3+^/Mg^2+^ cationic ratios within the garnet structure, combined with the controlled tuning of film thickness (ranging from 19 to 67 µm for CYMSSG:Ce/YAG and 10–22 µm for CYMSSG:Ce/YAG:Ce structures), enabled the precise modulation of their photoconversion properties. Prototypes of phosphor-converted WLEDs (pc-WLEDs) were developed based on these epitaxial structures to assess their performance and investigate how the content and thickness of SCFs affect the colorimetric properties of SCFs and composite converters. Clear trends were observed in the Ce^3+^ emission peak position, intensity, and color rendering, induced by the Y^3+^/Sc^3+^/Mg^2+^ cation substitution in the film converter, film thickness, and activator concentrations in the substrate and film. These results may be useful for the design of epitaxial phosphor converters with tunable emission spectra based on the epitaxially grown structures of garnet compounds.

## 1. Introduction

In recent years, the demand for high-efficiency, energy-saving light sources has significantly increased, driven by advancements in lighting and display technology. White light-emitting diodes (WLEDs) have emerged as the dominant choice due to their superior energy efficiency, long lifespan, and environmental benefits, including reduced carbon emissions and lower energy consumption compared to traditional incandescent and fluorescent bulbs [1]. Additionally, WLEDs have found applications beyond general lighting, such as in automotive headlights, backlighting for displays, and even in medical devices, further fueling the need for continuous improvements in their performance and efficiency [2,3].

A critical factor in the overall performance of WLEDs is the phosphor conversion layer, which plays a central role in color rendering and light quality. In most WLED configurations, a blue LED chip is combined with a phosphor material that converts a portion of the blue light into yellow, red, or green light, resulting in a balanced white emission. The efficiency of this conversion process directly impacts the brightness, color rendering index (CRI), and color correlated temperature (CCT) of the emitted light, all of which are crucial parameters for various applications [4,5,6].

The choice of phosphor material is therefore essential in the performance of the device. Among the various phosphor materials available, garnet-based materials doped with rare-earth ions, especially Ce^3+^, have proven to be among the most effective due to their broad excitation and emission spectra, high quantum efficiency, excellent luminescent properties, and thermal stability under high-power operation [7,8,9]. In particular, the Ce^3+^-doped Y_3_Al_5_O_12_ (YAG) phosphor is widely used in the industry for producing the high efficiency and good color quality of white light. It is compatible with blue LEDs and offers advantages such as strong absorption in the blue region, stable emission characteristics, and high thermal stability, which is crucial for maintaining performance over long operating hours [10].

The most common pc-WLEDs are produced using the Volume-Casting-Conversion (VCC) design, where a blue LED and powder PC are packed with organic resins [11]. Numerous techniques, including solid-state reactions [12], hydrothermal synthesis [13], coprecipitation [14], spray pyrolysis [15], sol–gel [16], and combustion [17], can be used to make ceramic powder phosphors. The heat generated from the LED chip and PC (Stokes shift and optical losses) cannot be efficiently dissipated because of the poor thermal stability and weak thermal conductivity of the resin (<0.5 W m^−1^ K^−1^) [1]. With the increasing demand for high-brightness lighting, the high-power wLEDs and white laser diodes (WLDs) are rapidly developing, and some limitations arise that are associated with the optical, structural, mechanical, and thermal properties of converters. The operating temperature of a color converter can reach even up to 200 °C under high-power LED or laser diode excitation. The extreme heat is generated in the converter, originating from the non-radiative relaxation of phosphors and the heat transfer from high-power LED chips. With the increase in the wLED temperature, color degradation occurs because of the thermal quenching properties of the phosphor, such as luminous decay and color shifting [18]. Furthermore, this setup results in low efficiency because the diffuse phosphor reflects 60% of the total white light back onto the chip [19]. To achieve high efficiency and strong chemical and thermal stability for pc-WLEDs, downconversion phosphors have been developed from powders to plates for the Planar-Chip-Level Conversion (PCLC) approach [20].

In PCLC design, instead of powder, the ceramic, eutectics, or single-crystal phosphors based on the yellow-emitting Ce^3+^-doped garnets are used for manufacturing high-power WLEDs under blue LED excitation. Nevertheless, glasses and ceramics have a relatively low luminescence efficiency [21], whilst the structure and optical properties of eutectic converters are hard to control. The development of single crystalline (SC) converters is much preferable to ceramics or powders, due to their higher uniformity and internal quantum efficiency (QE) than ceramics or glasses, as well as excellent thermal stability up to 300 °C, and high thermal conductivity (~10 W/(m K) [22,23,24].

It is obvious that the investigation of new types of color converters is an acute problem to be solved with the development of energy-efficient solid-state lighting sources. The minimization trend has focused attention on phosphor films that can be produced using different techniques such as sputtering deposition [25], electrochemical synthesis [26], pulsed laser deposition (PLD) [27], sol–gel [28], metal–organic chemical vapor deposition (MOCVD) [29], and liquid phase epitaxy (LPE) as well [30,31]. Among the presented methods, the LPE technique is a versatile method for the production of SCFs for applications in optoelectronics, with thicknesses in the range of several micrometers up to 200 μm, with excellent material quality and reproducibility [32].

One of the first works in this area was by Kundaliya et al. [32] and Markovskiy et al. [33], who proposed a phosphor converter based on the YAG:Ce and LuAG:Ce garnet phosphor film epitaxially grown onto a YAG substrate, to induce yellow and green emission, respectively. Recently, the possibility of the development of Tb_3_Al_5_O_12_:Ce (TbAG:Ce) single crystalline film converters for wLEDs using the LPE technique was shown [34]. It is especially important to note that, according to the Al_2_O_3_-Tb_2_O_3_ phase diagram, the Tb_3_Al_5_O_12_ melts incongruently, and it is difficult to grow a high-quality and large-size bulk TbAG:Ce crystal using Czochralski (Cz) or other melt-grown techniques, which is a barrier to its practical applications [35]. However, the single crystalline Tb_3_Al_5_O_12_ matrix or Tb_1.3_Gd_1.5_Al_5_O_12_ solid solution on their base can be fabricated in the pure garnet phase using low-temperature synthesis methods such as LPE growth [34,36].

The next generation of film converters is the development of a composite color converter (CCC), based on Ce^3+^-doped film/crystal epitaxial structures [36]. The development of CCCs suggests a few more additional tunable parameters, originating from the single-crystal substrate: (i) Ce^3+^ doping concentration in the substrate; (ii) thickness of the substrate. Finally, the combination of the emission coming from the Ce^3+^-doped substrate and the film constituents of the color converter allows for a wide spectrum of WLEDs similar to the spectrum of natural white light to be obtained, with enhanced luminous efficacy in comparison with standard photoconverters.

Phosphors based on the Ce^3+^-doped mixed {Ca_2_R}[Sc,B](C,Si_2_)O_12_; R = Lu, Y, Gd; B = Sc, Ga, C = Ga, Al silicate garnets can also be used for producing high-power WLEDs with a high color rendering index and low correlated color temperature values [37,38]. Due to the flexibility of the garnet structure, which allows for replacing ions at the dodecahedral { }, octahedral [ ], and tetrahedral ( ) sites, it is possible to replace the host cations and modify the conventional {Y}_3_[Al]_2_(Al)_3_O_12_ garnet composition for altering the Ce^3+^ spectroscopic properties to better meet the requirements for utilization in WLEDs. To date, the spectroscopic properties of Ce^3+^ in some garnets containing Si^4+^ at tetrahedral sites, namely Y_3_Mg_2_AlSi_2_O_12_, Y_3_MgAl_3_SiO_12_, CaY_2_Al_4_SiO_12_, MgY_2_MgAl_2_Si_2_O_12_, CaLu_2_Al_4_SiO_12_, CaLu_2_Mg_2_Si_3_O_12_, CaY_2_ZrSc Al_3_O_12_:Ce, and Lu_1.5_Ca_1.5_Al_3.5_Si_1.5_O_12_:Ce garnets, have been investigated [39,40,41,42,43,44,45,46,47,48,49,50,51].

It has been shown that Ca_3_Sc_2_Si_3_O_12_:Ce (CSSG) exhibits less thermal quenching of Ce^3+^ luminescence than YAG:Ce [37,50]. Mixed silicate garnets like Y^3+^-Mg^2+^ co-doped CSSG:Ce, e.g., (Ca,Y)_3_(Mg,Sc)_2_Si_3_O_12_ (CYMSSG), doped with Ce^3+^ ions, have shown also great potential as powder phosphor materials due to their broader emission spectra and absorption in the blue region than GSSG:Ce, which aligns well with the emission of blue LEDs [51,52]. However, despite the promising properties of these materials, research on the use of CYMSSG:Ce in the single crystalline films (SCF) [53] or crystals [54] as phosphor converters is limited, with even less focus on their performance in composite film–crystal structures based on the silicate garnets [55,56]. This gap hinders a complete understanding of how these materials behave under real LED excitation conditions and how variations in film structure, particularly thickness and variable cation content, influence their photoconversion efficiency.

The present study addresses this gap by investigating the structural, luminescent, and photoconversion properties of epitaxial converters based on Ce^3+^-doped CYMSSG-based SCFs with varying cation contents. These films were crystallized using the liquid phase epitaxy (LPE) method onto YAG and YAG:Ce single-crystal (SC) substrates, allowing for precise control over the growth process and material properties [18,19,20,21,22,23,24]. By systematically varying the film’s cation composition and thickness, ranging from 19 to 67 µm for Ce^3+^-doped CYMSSG films on YAG crystals (Series A) and from 10 to 22 µm for those on YAG:Ce crystals (Series B), we thoroughly examined how these parameters influence photoconversion efficiency.

This systematic approach enabled the observation and mapping of distinct trend lines in a chromaticity or color coordinate diagram, clearly illustrating the relationship between film content, thickness, and photoconversion performance. Variations in content and thickness affected key parameters, such as emission spectra and color rendering, which are critical for optimizing the overall luminescence and efficiency of the converter. These trend lines provide valuable insights into the optimal content and film thickness required for enhanced photoconversion, highlighting the importance of precise control over all SCF parameters to achieve the desired optical characteristics in phosphor converters for WLED applications.

## 2. SCF Growth

Two distinct sets of thin films with nominal compositions of Ce^3+^-doped Ca_2_YMgScSi_3_O_12_ and Ca_1.75_Y_1.25_Mg_1.25_Sc_0.75_Si_3_O_12_ (Series A and B, respectively), with thicknesses ranging from 10 µm to 67 µm, were fabricated using the LPE method (Table 1). The films were crystallized within a temperature range of 975–990 °C from a supercooled melt solution composed of a PbO:B_2_O_3_ flux with a mole ratio of 12:1. The CYMSSG:Ce SCFs were grown on undoped YAG substrates for Series A and on Ce^3+^-doped YAG substrates for Series B, with orientations close to the (111) crystallographic plane. The YAG and YAG:Ce substrates used in these experiments had a thickness of 0.5 mm. The nominal Ce concentration in the CYMSSG:Ce SCFs and YAG:Ce substrates was approximately 0.05–0.15 at. % and 0.05–0.06 at.%, respectively (Table 1). Further details about the growth process for Ca-Si-based films and the specific mole ratios used for LPE growth can be found in references [57].

The thickness of the SCF samples, denoted as **h** (in μm), was determined using a weighing method. This approach involved measuring the substrate’s mass before and after the SCF growth cycle with high-precision scales. Film thickness was then calculated using the following formula: **h = (m − m_s_)/(2 × S × ρ),** where **m** is the mass of the substrate with the grown SCF (in grams), **m_s_** is the mass of the substrate (in grams), **S** is the substrate area (in cm^2^), and **ρ** is the film density (in g/cm^3^).

The compositions of single crystals and films were analyzed using a JEOL JSM-820 electron microscope (JEOL Ltd., Akishima, Japan) equipped with an IXRF 500i LN_2_ Eumex EDX detector (IXRF, Inc., Austin, TX, USA). This advanced tool provided rapid assessments and ensured the precise detection of elemental variations with an accuracy of ±1%. The analysis revealed considerable deviations in the concentrations of Ca, Mg, Sc, and Si cations compared to the nominal (in-melt) formulas (Ca,Y)_3_(Mg,Sc)_2_Si_3_O_12_ and Ca_1.75_Y_1.25_Mg_1.25_Sc_0.75_ Si_3_O_12_:Ce. Meanwhile, these deviations were consistently less than ±0.2 formula units, as shown in Table 1. Such findings underscore the importance of precise compositional control in material synthesis. To maintain clarity and alignment with experimental results, the study consistently refers to the nominal composition of the SCFs. This approach ensures a standardized comparison while accounting for the slight variations observed during the evaluation process.

**Figure 1:**
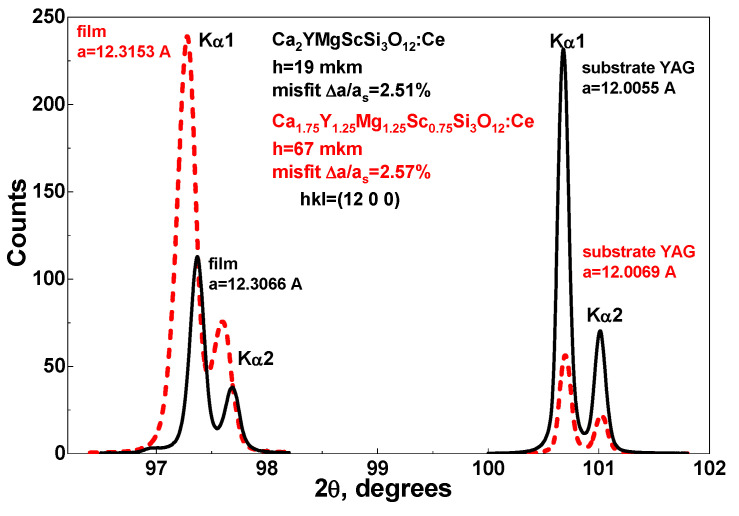
XRD patterns of (12 0 0) plane of Ca_2_YMgScSi_3_O_12_:Ce SCF (sample B1) and Ca_1.75_Y_1.25_Mg_1.25_Sc_0.75_Si_3_O_12_:Ce SCFs (sample B4) grown on YAG substrate.

The structural quality of Ca_2−X_Y_1₊X_Mg_1₊X_Sc_1−X_Si_3_O_12_:Ce (x = 0–0.25) films grown on YAG substrates was investigated using X-ray diffraction (XRD) with a DRON 4 spectrometer (Saint-Petersburg, former USSR) equipped with a Cu_Kα_ X-ray source. For this analysis, Samples B1 and B4 with x = 0 and x = 1.25 were chosen, featuring a 0.25 Ce^3+^ concentration of 0.01 and 0.15 at.% and a thickness of 19 µm and 67 µm, respectively, as illustrated in Figure 1 and Table 1. This sample was selected for its suitability for detailed structural evaluation.

Typically, the single crystallinity of CYMSSG films grown on YAG substrates is confirmed using thinner samples, generally with thicknesses of less than 50–70 µm. This preference is driven by the inherent limitations associated with X-ray diffraction in thicker samples. Specifically, the garnet matrix of CYMSSG films exhibits medium X-ray absorption, which changes the relative intensity of diffraction reflections from the YAG substrate. This effect becomes more pronounced as the film thickness increases, complicating accurate structural characterization in thicker samples (Figure 1).

Therefore, thinner samples are prioritized for initial analysis to reliably confirm single crystallinity. Once verified, the growth process continues to produce thicker films (exceeding 100 µm) for further experimental studies. This sequential approach ensures that the structural integrity and quality of the CYMSSG films are thoroughly assessed before the fabrication of thicker film samples. By addressing the technical constraints of XRD analysis, this method provides a robust framework for characterizing the structural properties of CYMSSG films on YAG substrates, paving the way for their application in advanced optical and photonic systems.

The lattice misfit between the CYMSSG SCFs and the YAG substrate was calculated using the XRD pattern corresponding to the (12 0 0) crystallographic plane of the sample (Figure 1). The standard formula for lattice misfit, expressed as Δa = ((a_SCF_ − a_sub_)/a_sub_) × 100%, was used, where **a_SCF_** is the lattice parameter of the film, and **a_sub_** is the lattice parameter of the YAG substrate. The analysis revealed a lattice mismatch of approximately 2.51–2.57%, which indicates a significant mismatch between the SCF and substrate lattices. Meanwhile, even this degree of mismatch was found to be consistent with high-quality epitaxial growth, where strain and stress at the interface are not yet critical, maintaining the crystalline integrity of the film. This finding is particularly significant as it confirms the successful epitaxial growth of the CYMSSG (x = 0–0.25) film on the YAG substrate.

## 3. Experimental Technique

To comprehensively investigate the properties of the two sets of thin-film samples (Series A and B) and Ce-doped substrates (Series C), an extensive suite of spectroscopic techniques was employed. These techniques included absorption spectroscopy, photoluminescence (PL) emission, and excitation (PLE) spectroscopy, which together provided a detailed evaluation of the luminescent characteristics of the CYMSSG:Ce SCFs. All spectroscopic measurements were conducted under ambient conditions at room temperature (RT), ensuring their relevance to practical applications.

The absorption spectra of the SCFs were recorded using a Jasco V730 spectrophotometer (Tsukuba, Japan). For a detailed examination of the photoluminescent properties, an FS-5 spectrometer (Edinburgh Instruments, Livingston, UK) was used. Photoconversion spectra were recorded using an AvaSpec-ULS 2048-LTEC fiber-optic spectrophotometer, paired with an AvaSphere-50-IRRAD integrating sphere (Avantes, Apeldoorn, The Netherlands). These measurements enabled a comprehensive analysis of the SCFs’ photoconversion performance, shedding light on their potential for advanced photonic applications. The Osram LBE 6SG (I_f_ = 30 mA, V = 2.9 V) blue LED 455 nm (30 mA, 2.9 V) was used as an excitation source to determine the chromaticity parameters of the samples.

## 4. Absorption and Luminescent Properties

### 4.1. Absorption Spectra

**Figure 2:**
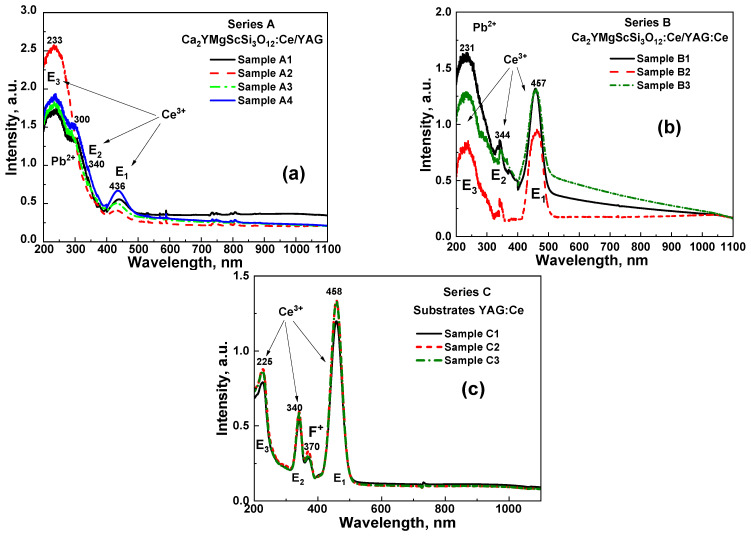
RT absorption spectra of CYMSSG:Ce SCF/YAG (**a**) and CYMSSG:Ce SCF/YAG:Ce (**b**) in comparison with YAG:Ce substrates (**c**).

The absorption spectra of the CYMSSG:Ce SCF/YAG SCFs (Figure 2a) and CYMSSG:Ce SCF/YAG:Ce (Figure 2b) composite samples, as well as the absorption spectra of the YAG:Ce SC substrates (Figure 2c), exhibit distinct features in the 200–500 nm wavelength range, reflecting their optical properties. Notably, broad absorption bands peaking around 341 nm and within the 439–457 nm range, referred to as E2 and E1 bands, respectively, are consistently observed across all analyzed structures, confirming their successful incorporation into the CYMSSG films. These bands are characteristic for the absorption of Ce^3+^ ions in the garnet host, corresponding to the 4f^1^(^2^F_5_/_2_)→5d (^2^E) electronic transitions. Additionally, a spectral feature at 230 nm is attributed to the 4f (^2^F_5_/_2_)→5d_1_ (T_2g_) transitions of Ce^3+^ ions (E_3_ band). These identifications align with prior studies, which highlight these transitions as key spectral signatures of Ce^3+^ ions’ presence in garnet matrices [1,2].

Another notable feature of SCF samples is an absorption bands peaked at 300 nm and below 220 nm, corresponding to the ^1^S_0_→^3^P_1_ and ^1^P_1_ electronic transitions of Pb^2+^ impurity, likely introduced into the film during LPE growth from PbO-based flux. The last Pb^2+^-related band strongly overlapped with the E_3_ band of Ce^3+^ ions. Despite being associated with trace Pb^2+^ inclusions, this feature provides valuable insights into the real material’s composition and the interactions between dopants and Ce^3+^ ions [3].

In addition to these primary features, low intensive absorption bands in the UV range, peaking at 370 nm, are evident in the YAG:Ce substrates and absorption spectra of the CYMSSG:Ce SCF/YAG:Ce structure. Generally, such UV bands have been attributed to defect center absorption in garnets, grown or annealed in the reducing atmosphere [58], or containing an excess of Ca^2+^ ions [53,57], and correspond to the absorption of the F^+^ center (one charged anion vacancy).

Overall, the analysis of these absorption spectra confirms the effective doping of the SCFs with Ce^3+^ ions and underscores the impact of trace impurities on their optical behavior.

### 4.2. PL Spectra

**Figure 3:**
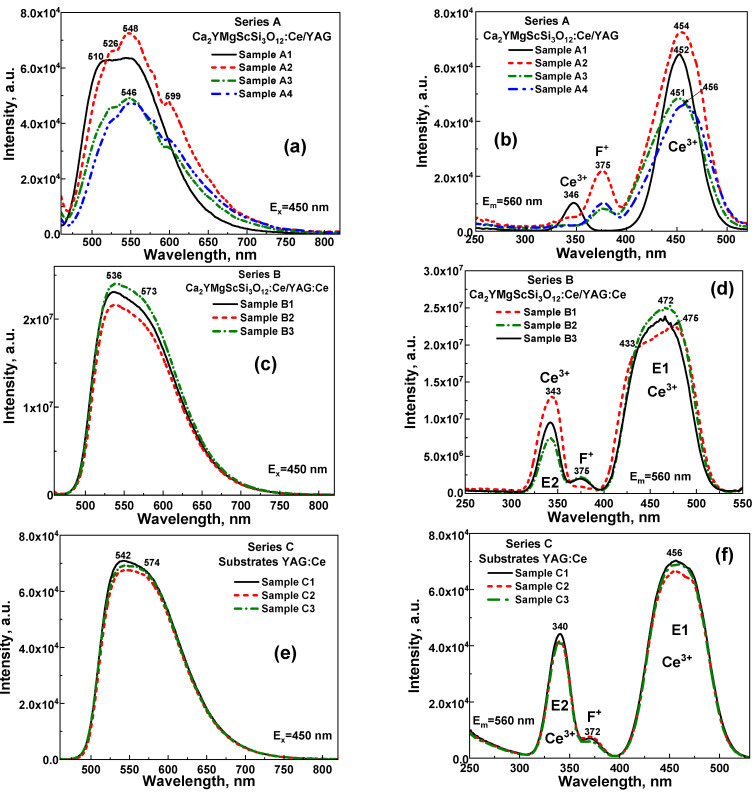
RT PL (**a**,**c**,**e**) and PLE (**b**,**d**,**f**) spectra of CYMSSG:Ce SCF/YAG ((**a**,**b**)—A-series) and CYMSSG:Ce/YAG:Ce ((**c**,**d**)—B series) structures in comparison with YAG:Ce substrates ((**c**)—series C).

The PL and PLE spectra of samples from Series A and B, as well as substrates from Series C, are shown in Figure 3. The PL spectra of all SCF samples (Figure 3a,b) exhibit intense luminescence characterized by broad emission bands in the green-yellow spectral range. These bands are attributed to the 5d_1_→4f (^2^F_5_/_2_, ^2^F_7_/_2_) electronic transitions of Ce^3+^ ions, which are characteristic of the luminescent behavior of cerium-doped garnets. However, it should be noted that Ce^3+^ emission bands consist of several sub-bands corresponding to non-equivalent Ce^3+^ multicenters. Specifically, Ce^3+^ ions replace the dodecahedral positions of Ca^2+^ and Y^3+^ cations, which have different local surroundings with Mg^2+^ and Sc^3+^ ions in octahedral positions and Si^4+^ in tetrahedral positions within the garnet lattice. The observed structure of the PL spectra of CYMSSG:Ce SCFs grown on a YAG substrate clearly indicates the presence of multiple Ce^3+^ emission centers in the CYMSSG host, as described in detail in our previous works [8,18].

The PL spectrum of samples from Series A, which were grown on an undoped YAG substrate, exhibits a broader Ce^3+^ luminescence band, with two distinct peaks at 510 nm and 548 nm. The slight blue shift in the PL emission and excitation spectra of sample A1 is caused by its lower cerium concentration, which is three times lower than that of the other samples in this set. This blue shift occurs because the reduced Ce^3+^ concentration limits the energy transfer between different Ce^3+^ multicenters, primarily affecting those emitting in the longer-wavelength range.

Conversely, the PL spectra of samples from Series B, grown on a Ce^3+^-doped YAG substrate, display a notably broader emission band with peaks at 536 nm and 573 nm due to the overlap between the PL spectra of the YAG:Ce substrate (Figure 3c) and the CYMSSG:Ce SCFs (Figure 3a). This red shift may also result from variations in the crystal field strength or changes in the covalency of Ce^3+^–ligand bonds within the Ca^2+^-Si^4+^-based CYMSSG host compared to the YAG:Ce substrate [8,18]. Specifically, increased covalency in Ce^3+^–ligand bonds within the CYMSSG matrix reduces the energy required for Ce^3+^ electronic transitions, contributing to the observed red shift.

Interactions between Ce^3+^ ions in YAG:Ce substrates and CYMSSG:Ce films may also lead to energy transfer processes that influence the emission spectra of the epitaxial structure. Understanding these wavelength shifts is crucial for tailoring the material’s properties and optimizing its emission for specific applications. These findings highlight the critical role of the substrate composition in determining the photoluminescence properties of CYMSSG:Ce films. The interactions between SCFs and substrates, particularly those involving cerium doping, significantly impact the energy transfer dynamics and luminescent behavior.

### 4.3. PLE Spectra

The PLE spectra of Ce^3+^ ions in YAG:Ce substrates and CYMSSG SCF/YAG and CYMSSG SCF/YAG:Ce structures are presented in Figure 3b,d,f. The 4f (^2^F_5_/_2_)→5d transitions are key characteristics of Ce^3+^ ion excitation and play a fundamental role in its luminescent behavior. The peak near 456 nm in YAG:Ce substrates is typically associated with the transition from the ground state to the lowest 5d energy level, whereas the 340 nm peak corresponds to a transition to a higher-lying 5d state. These transitions are governed by the symmetry and electronic environment of Ce^3+^ ions within the garnet matrix. The relative intensities and positions of these bands provide insights into the local crystal field and the energy splitting of the 5d states.

Furthermore, the peaks at 340 nm and 456 nm indicate that the YAG:Ce substrate and its composite structure exhibit efficient absorption capabilities at multiple wavelengths, enabling excitation in both the UV and visible regions. This dual-excitation feature enhances the material’s photoluminescent properties, making it suitable for applications in lighting, displays, and optoelectronic devices.

The PLE spectra for Series A (Figure 3b) exhibit prominent maxima at 346 nm and 454 nm, corresponding to the 4f→5d electronic transitions of Ce^3+^ ions in the CYMSSG host. These peaks are typical of cerium-doped materials and indicate efficient absorption in the UV and blue regions, making them suitable for excitation by respective LEDs.

The PLE spectra of samples from Series B (Figure 3d) represent a superposition of the PLE spectra of the YAG:Ce substrate and CYMSSG:Ce SCF. These spectra feature a peak at 343 nm (E_2_) and a broad complex excitation band in the blue region centered at 472 nm (E_1_). The latter band is a superposition of at least two distinct peaks located at 433 nm and 476 nm. The presence of multiple peaks within the E_1_ band is typically attributed to the excitation of Ce^3+^ multicenters in the CYMSSG:Ce film [8,18].

The excitation peak observed at 375 nm in the PLE spectra of Ce^3+^ luminescence in CYMSSG:Ce SCFs is closely related to intrinsic electronic transitions associated with F^+^ centers in garnets [1,17]. Specifically, this peak corresponds to the ^1^A→^1^B transition of the F^+^ center, a well-known defect in many crystalline materials, including oxides. The F^+^ center refers to an oxygen vacancy typically associated with a trapped electron. The presence of this excitation band at 375 nm suggests that F^+^ centers play a role in the optical behavior of CYMSSG:Ce SCFs, contributing to the material’s overall photonic properties. In particular, the material’s ability to absorb light at this wavelength may influence the efficiency of energy transfer processes and the overall photoluminescent behavior of Ce^3+^ ions, as interactions between F^+^ centers and Ce^3+^ ions can modify emission characteristics.

## 5. Photoconversion Properties

Prototypes of phosphor-converted white light-emitting diodes (pc-WLEDs) were developed to assess their performance and to investigate how the content and thickness of SCFs affect the colorimetric properties of SCFs and composite converters. These pc-WLEDs were assembled by integrating epitaxial SCFs and composite converters into the OSRAM LBE 6SG blue LED chip (ams OSRAM AG, 8141, Premstaetten, Austria) with a peak emission wavelength of 450 nm. These chips operated at a fixed forward bias voltage of 2.6 V and a drive current of 20 mA.

**Figure 4:**
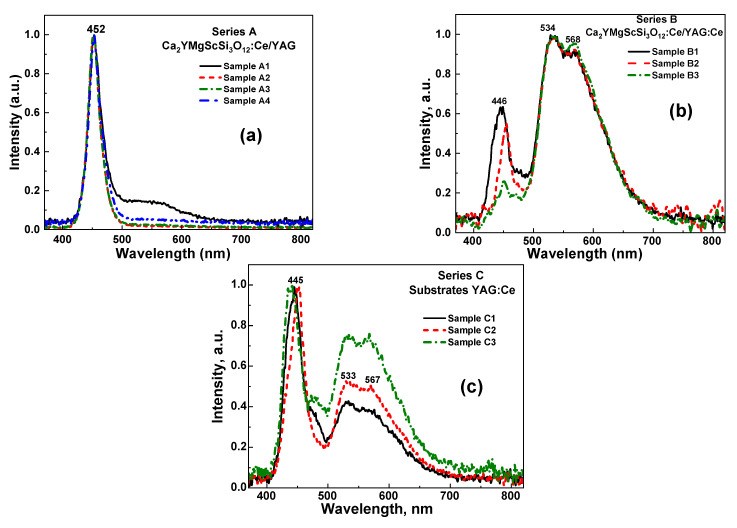
Normalized emission spectra of pc-WLED prototypes fabricated on the base of 450 nm LED chip and CYMSSG:Ce SCF converters grown on YAG (**a**) and YAG:Ce (**b**) substrates in comparison with converters on the base of YAG:Ce crystal substrates (**c**).

The emission spectra of the prototypes, presented in Figure 4, correspond to converters containing CYMSSG:Ce SCFs with varying contents and thicknesses, grown on undoped (Series A) and Ce^3+^-doped (Series B) YAG substrates, each with a thickness of 0.5 mm (see Table 1 for details). For comparison, an emission diagram of the YAG:Ce substrate (Figure 4c), used as a conventional reference sample, is also provided alongside the corresponding diagram of the composite CYMSSG:Ce/YAG:Ce structures (Figure 4b).

The emission spectrum generated by these white LED prototypes demonstrates a clear dependence of Ce^3+^ emission intensity on the thickness of the CYMSSG:Ce SCF (Figure 4a,b). As the film thickness increases, a larger number of Ce^3+^ ions are excited by the incident blue light, resulting in the stronger absorption of the blue component. This, in turn, enables the tailoring of the blue-to-yellow emission ratio (Figure 4a,b). Specifically, the addition of emission from the CYMSSG:Ce SCFs significantly reduces the blue component and increases the yellow and red components in the total emission of the composite prototypes, compared to conventional YAG:Ce crystal converters.

**Figure 5:**
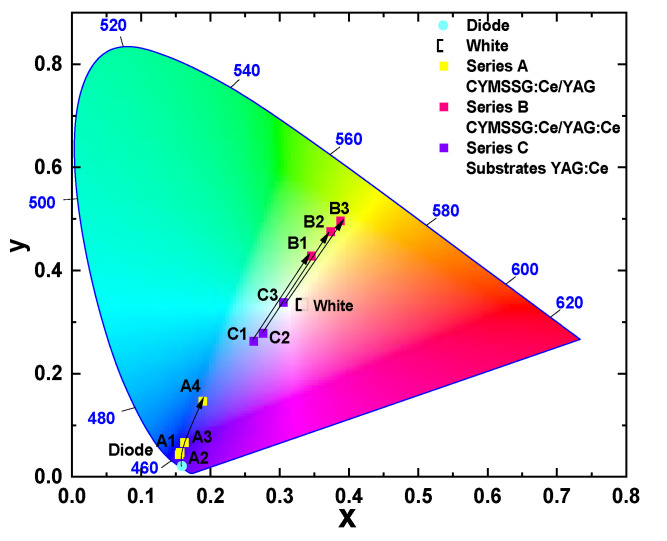
Chromaticity diagram of a WLED prototype fabricated on the base of 450 nm LED chip and CYMSSG:Ce SCFs grown on YAG (Series A) and YAG:Ce (Series B) substrates. The results for YAG:Ce substrates are presented for comparison.

For a more detailed analysis, the emission of WLED prototypes is plotted also in a CIE diagram (Figure 5). Table 2 presents the CIE chromaticity coordinates, color rendering index (CRI), correlated color temperature (CCT), and luminous efficiency (LE) of the developed WLEDs, highlighting their effectiveness in achieving desirable lighting characteristics. These parameters are critical for evaluating the lighting performance of the developed prototypes, providing insight into their color accuracy, spectral balance, and suitability for various applications.

CYMSSG:Ce SCF samples from Series A, grown on undoped YAG substrates, exhibited emission coordinates clustered within the blue region of the chromaticity diagram (Figure 5). Coordinates corresponding to the YAG substrates were not included here, as these substrates do not contribute to photoconversion and their emission characteristics remain identical to those of the original blue diode. This indicates that the photoconversion behavior of the CYMSSG:Ce SCF/YAG structures is determined solely by the properties of the films, without additional contributions from the substrate. Meanwhile, the conversion efficiency of these films, with thicknesses ranging from 19 to 50 μm, is low, and only the 67 μm-thick SCF A4 sample shows a clear visible trend in CIE coordinate change with increasing thickness (yellow squares in Figure 5).

The coordinates of the YAG:Ce crystal substrates (blue squares) are also plotted on the diagram as reference samples for comparison with film and composite converters (Figure 5). As shown in Figure 5, the color coordinates of the YAG:Ce crystal converters are significantly influenced by the Ce^3+^ concentration. In particular, the YAG:Ce crystal, with 0.075% Ce^3+^ content (sample C3) and a thickness of 0.5 mm, exhibits CIE coordinates of (x = 0.305, y = 0.338) and a correlated color temperature (CCT) of 6840 K, which are very close to the standard white light reference. In contrast, samples C1 and C2, with lower Ce^3+^ contents (0.05%), show color coordinates that fall within the sky-blue region. Moreover, increasing the Ce^3+^ content in the YAG:Ce crystal converters results in a slight decrease in the CRI value, but a notable improvement in luminous efficiency from 71 to 72 for samples C1 and C2, and to 79 for sample C3 (Table 2).

In contrast, CYMSSG:Ce SCF/YAG:Ce composite converters (Series B) exhibited emission coordinates in the green-yellow region (red squares). This pronounced shift is primarily attributed to the presence of Ce^3+^ dopant in the SCF converters, which introduces additional photoluminescent features, thereby enhancing the overall emission and significantly altering the photoconversion behavior of the epitaxial structures. In this case, the color coordinates and CCT are less dependent on the properties and Ce^3+^ content of the underlying crystal substrates. Instead, the composition of the SCF converters, specifically, the ratio of Y/Mg/Sc cations as well as the film thickness, play a more dominant role in determining the photoconversion characteristics of the composites (Figure 5). These parameters can be effectively tuned to optimize the performance of the fabricated WLEDs. Notably, as the SCF thickness increases, the composite converters show a reduction in the color rendering index (CRI), with values in the range of 59–62. However, they demonstrate a significantly higher luminous efficiency, reaching 102–115 lm/W, compared to the corresponding YAG:Ce substrates (Table 2), primarily due to the enhanced absorption and emission properties of both the film and substrate converters. The highest luminous efficacy achieved is 115 lm/W for sample S3.

Thus, the proposed approach for creating composite color converters demonstrates the ability to achieve virtually any desired color coordinates within the white light region by adjusting several key parameters: (i) the Ce^3+^ concentration in the YAG:Ce substrate, (ii) the film composition, and (iii) the film thickness. The results also highlight the critical role of both the film and substrate composition, as well as the SCF thickness, in determining the photoconversion performance of these composite converters. By carefully tuning these parameters, it is possible to tailor the colorimetric properties of pc-WLEDs to meet the specific requirements of various white solid-state lighting applications. This study illustrates the potential of CYMSSG:Ce SCFs as highly versatile and effective materials for next-generation pc-WLED technologies, paving the way for further advancements in the field.

## 6. Conclusions

This study investigates the structural, luminescent, and photoconversion properties of Ce^3+^-doped film–crystal composite converters based on epitaxial structures containing Ce^3+^-doped single crystalline films (SCFs) of Ca_2−x_Y_1+x_Mg_1+x_Sc_1−x_Si_3_O_12_:Ce (x = 0–0.25) (CYMSSG:Ce), grown using the liquid phase epitaxy (LPE) method on Y_3_Al_5_O_12_ (YAG) and YAG:Ce substrates. For this purpose, two series of CYMSSG:Ce SCFs with different ratios of Y, Mg, and Sc cations and varying thicknesses in the 19–67 µm range were synthesized on YAG and YAG:Ce substrates.

X-ray diffraction (XRD) analysis confirmed the presence of the epitaxial growth of high-quality SCFs, revealing a lattice misfit between the CYMSSG:Ce SCF and the YAG substrate in the range of 2.51–2.57%. Absorption spectra exhibited broad bands around 340 nm and 436–458 nm, characteristic of Ce^3+^ 4f–5d transitions, confirming the successful doping of CYMSSG:Ce SCFs. A weak absorption band below 300 nm suggests trace Pb^2+^ impurities resulting from the LPE growth process using a PbO-based flux.

Photoluminescence (PL) measurements of Ca_2−x_Y_1+x_Mg_1+x_Sc_1−x_Si_3_O_12_:Ce showed broad green-yellow emission bands due to Ce^3+^ transitions. Increasing the Y and Mg content (**x**) in the films led to a red shift in the Ce^3+^ emission spectra, while the SCF of this garnet with a large Sc content demonstrated a more pronounced blue shift of Ce^3+^ luminescence. Excitation spectra revealed prominent peaks at 340 and 450 nm related to the 4f-5d^1,2^ Ce^3+^ transitions, while a peak near 375 nm was attributed to F^+^ centers.

Prototype phosphor-converted white LEDs (pc-WLEDs) were fabricated using CYMSSG:Ce SCF/YAG substrate and CYMSSG:Ce SCF/YAG:Ce substrate structures with various SCF thicknesses placed directly onto blue-emitting InGaN chips. We found that films grown on undoped YAG substrates (Series A) displayed very low conversion efficiency, while the CYMSSG:Ce SCF/YAG:Ce substrate structures (Series B) exhibited promising characteristics for white LED applications. Chromaticity analysis of the latter structures demonstrated that both the YAG:Ce substrate and the CYMSSG:Ce SCF with different contents and thicknesses significantly affect the photoconversion performance of the WLED prototypes and can be used for the effective tuning of the tone of white light on demand. Namely, increasing the SCF thickness results in a slight decrease in the CRI value, but a notable improvement in the luminous efficiency of composite converters. The highest luminous efficacy of 115 lm/W is achieved for the Ca_1.75_Y_1.25_Mg_1.25_ Sc_0.75_Si_3_O_12_:Ce SCF (22 μm)/YAG:Ce (0.12%) substrate composite sample.

## Figures and Tables

**Table 1:** The nominal composition (in melt solution) and the measured (using EDX) composition of LPE-grown CYMSSG:Ce/YAG and CYMSSG:Ce/YAG:Ce films (Series A and B, respectively) and YAG:Ce substrates (Series C).

Sample	Nominal Content in the Melt	Measured SCF Composition	h, µm
A1	Ca_2_YMgScSi_3_O_12_:Ce	Ca_1.88_Y_1.09_Ce_0.01_Mg_0.9_Sc_1.42_Si_2.73_O_12_	19
A2	Ca_2_YMgScSi_3_O_12_:Ce	Ca_1.83_Y_1.08_Ce_0.03_Mg_0.9_Sc_1.51_Si_2.68_O_12_	34
A3	Ca_2_YMgScSi_3_O_12_:Ce	Ca_1.81_Y_1.12_Ce_0.03_Mg_0.93_Sc_1.48_Si_2.67_O_12_	49
A4	Ca_1.75_Y_1.25_Mg_1.25_Sc_0.75_Si_3_O_12_:Ce	Ca_1.63_Y_1.27_Ce_0.03_Mg_1.19_Sc_1.27_Si_2.72_O_12_	67
B1	Ca_2_YMgScSi_3_O_12_:Ce	Ca_1.92_Y_1.08_Ce_0.02_Mg_0.95_Sc_1.27_Si_2.78_O_12_	10
B2	Ca_1.75_Y_1.25_Mg_1.25_Sc_0.75_Si_3_O_12_:Ce	Ca_1.65_Y_1.35_Ce_0.03_Mg_1.18_Sc_0.61_Si_3.21_O_12_	11
B3	Ca_1.75_Y_1.25_Mg_1.25_Sc_0.75_Si_3_O_12_:Ce	Ca_1.68_Y_1.32_Ce_0.03_Mg_1.28_Sc_0.71_Si_3.01_O_12_	22
C1	Y_3_Al_5_O_12_:Ce	Y_2.99_Ce_0.01_Al_5_O_12_	500
C2	Y_3_Al_5_O_12_:Ce	Y_2.99_Ce_0.01_Al_5_O_12_	500
C3	Y_3_Al_5_O_12_:Ce	Y_2.988_Ce_0.012_Al_5_O_12_	500

**Table 2:** Comparison of the CIE coordinates, CRI, CCT, and LE of epitaxial structures based on the CYMSSG:Ce SCF samples grown on YAG (Series A, Series B) and YAG:Ce (Series C) substrates. The results for YAG:Ce substrates, as reference samples, are presented for comparison.

Samples	SCF Thicknesses, µm	Type and Thicknesses of Substrate, mm	CIE Coordinates		CCT, K	CRI	LE,lm/W
			y	y			
**Series A**							
A1	19	0.5	0.162	0.066		-	-
A2	34	0.5	0.155	0.043		-	-
A3	49	0.5	0.156	0.047		-	-
A4	67	0.5	0.189	0.146		-	-
**Series B**							
B1	10	C1; 0.5	0.346	0.428	5150	62	102
B2	11	C2; 0.5	0.374	0.475	4612	59	112
B3	22	C3; 0.5	0.388	0.496	4406	59	115
**Series C**							
C1	-	0.5	0.263	0.263	-	78	71
C2	-	0.5	0.276	0.278	-	75	72
C3	-	0.5	0.305	0.338	6838	70	79

## Data Availability

The original data presented in this study are included in the article as figures embedded in the Word version of the paper. These figures can be opened and accessed using any version of Origin software. For further inquiries, please contact the corresponding authors.
